# Dog Owners Exhibit Better Diet Quality but Similar Physical Activity Compared to Non-Owners: A Case-Control Study

**DOI:** 10.3390/nu18010078

**Published:** 2025-12-26

**Authors:** Konstantinos Lazaridis Margaritis, Marilena Perantonaki, Katerina Pyrga, Eleni C. Pardali, Dimitrios Poulimeneas, Dimitrios G. Goulis, Maria Tsigga, Maria G. Grammatikopoulou

**Affiliations:** 1 Department of Nutritional Sciences & Dietetics, Faculty of Health Sciences, International Hellenic University, Alexander Campus, Sindos, P.O. Box 141, GR-57400 Thessaloniki, Greece; 2 Department of Nutrition and Dietetics, Harokopio University, 70 El. Venizelou Avenue, Kallithea, GR-17671 Athens, Greece; 3 Immunonutrition Unit, Department of Rheumatology and Clinical Immunology, Faculty of Medicine, School of Health Sciences, University of Thessaly, Biopolis, GR-41223 Larissa, Greece; 4 Department of Nutritional Science and Dietetics, School of Health Sciences, University of the Peloponnese, Antikalamos, GR-24100 Kalamata, Greece; 5 Unit of Reproductive Endocrinology, 1st Department of Obstetrics and Gynaecology, Medical School, Aristotle University of Thessaloniki, GR-54124 Thessaloniki, Greece

**Keywords:** Fitbit, physical activity, CVD, body composition, nutrition, pet ownership, exercise, pedometers, companion animal, accelerometer, obesity, lifestyle medicine

## Abstract

**Introduction**: “The dog is a man’s best friend” and research has showed that this idea is extended beyond the degree of loyalty. Dog ownership has been linked to several positive health outcomes for the owner. The aim of the present cross-sectional case–control study was to assess differences in the physical activity level (PAL), body composition, quality of life (QoL), and diet quality and dietary knowledge between dog owners and non-owners. **Methods**: A total of 55 dog owners and an equal amount of non-dog owners (all aged between 18 and 60 years old) formed the case and control groups, respectively. Basic anthropometric measurements were performed, including body fat (BF) and diet, assessed with the Mediterranean Diet Score (MedDietScore) and the Eating Assessment Table (EAT). Physical activity was recorded for 3 consecutive days using activity monitors. QoL was evaluated using the brief version of the World Health Organization QoL (WHOQOL-BREF) tool. **Results**: The two groups demonstrated a similar PAL, but lower BF% (*p* = 0.009), hip circumference (*p* < 0.001), triceps (*p* = 0.012), and subscapular skinfolds (*p* = 0.003) were recorded among dog owners. The EAT score was greater among dog owners (*p* = 0.0023), indicating improved dietary intake and knowledge, even after adjustment for education attained and BMI (*p* = 0.026). On the other hand, greater adherence to the Mediterranean diet was exhibited among those not having dogs (*p* = 0.018). Regarding dog measurements and their owners’ anthropometry, dog neck circumference was negatively correlated to the owners’ biceps and triceps skinfolds (r = −0.327, *p* = 0.016; r = −0.320, *p* = 0.018, respectively). Additionally, dog breed size was negatively correlated to the owners’ triceps skinfold (r = −0.325, *p* = 0.015), sum of skinfolds (r = −0.311, *p* = 0.021), hip circumference (r = −0.341, *p* = 0.011), body fat (r = −0.357, *p* = 0.007), and fat mass index (r = −0.307, *p* = 0.023). **Conclusions**: Dog ownership is associated with improved body composition and smaller skinfold thickness at specific body sites, as well as with a more health-conscious lifestyle, including better diet quality and knowledge.

## 1. Introduction

An old universal quote considers the dog as man’s best friend, and research has shown that this extends beyond the degree of loyalty. The significance of animals in our lives stems from the close human–animal bond, a dynamic relationship that is mutually beneficial, supporting the health and well-being of both in this partnership [1]. A plethora of studies have associated dog ownership with reduced cardiovascular disease (CVD) outcomes [2,3,4,5], resulting in the release of a related scientific statement by the American Heart Association [6]. In parallel, research has revealed that pet ownership is associated with anti-hypertensive effects [7], reduced all-cause and CVD-related mortality [5,8], and a protective impact even among patients with established CVD [5].

Companion dogs are important components of the “social support” of their owners, strengthening their engagement in weight loss programs and increasing exercise motivation [9,10] and physical activity levels (PAL) [11,12,13]. Additionally, pet owners seem to report a better quality of life (QoL) and improved sleep quality compared to non-owners [14,15]. The majority of pet owners regard their pets as members of their family, highlighting the complexity of emotional bonds and subsequent effects on mental health [16,17]. Pets can also provide emotional and psychological support, particularly during periods of high stress. For instance, during the COVID-19 pandemic, companion animals exerted a positive impact on reducing depression, anxiety, isolation, and loneliness of their masters [18].

With obesity evolving to a global epidemic, researchers have confirmed this latter finding and suggested that dog walking might contribute to a more active lifestyle, and thus, dog ownership has been proposed for inclusion in the relevant Physical Activity Task Force [19]. Several studies have supported this theory, using accelerometers and activity PAL monitors to demonstrate that dog owners engage in increased low- and moderate-intensity physical activity [12,20,21]. Coleman and associates showed that dog owners were more likely to meet the national recommendations for moderate to vigorous physical activity (MVPA) and presented lower rates of obesity compared to non-owners [12]. Dall et al. found that dog owners reported fewer sitting events, apart from additional time spent walking their pets [21].

In Greece, a country with high obesity rates [22,23] and weather favorable for taking walks, the need for increased physical activity is considered a health priority, and pet adoption might enhance motivation for implementing this goal. Furthermore, pets and their owners appear to be associated beyond the extent of physical activity motivation, as studies have shown the existence of a relationship between pet and owner weight status, suggesting a strong immediate environmental effect, despite the absence of a shared genetic pool [24]. Research is consistent that dogs are most likely to become obese when their owners are overweight [25,26,27], as a possible result of mutual physical inactivity and sedentary behavior. In parallel, even dietary choices appear to be aligned between dog owners and their pets, with science suggesting that vegan dog owners tend to adjust their pets’ diet to a more plant-based prototype [28]. Despite this, no study has specifically examined the dietary knowledge of dog owners or whether dog ownership is associated with differences in diet quality.

The aim of the present case–control study was two-fold: (a) to assess PAL, nutritional knowledge, and diet quality, as well as QoL between dog owners and non-owners with the use of activity monitors, dietary indexes, and a QoL questionnaire, respectively, and (b) to define possible associations between the anthropometric characteristics of dog owners and their companion dogs.

## 2. Materials and Methods

### 2.1. Study Design

This study was conducted according to the guidelines of the Helsinki Declaration, and all procedures were approved initially by the Alexander Technological Educational Institute and also by the Ethics Committee of the University of Thessaly (164th/5 December 2025). Informed consent was obtained from all subjects.

A total of 57 adults, aged between 18 and 64 years old, all dog owners, were recruited from social media, veterinary clinics, and dog parks in Thessaloniki, forming the cases group. An equal number of adults not owning a dog were randomly recruited from an advertisement on social media during the same period and formed the control group. Recruitment took place during June–November 2015. The recruited dog owners were all responsible for walking their dog within their family. Two participants from each group were removed from the analyses for failing to complete the physical activity tracker and not being responsible for walking their dogs, respectively; thus, the remaining sample consisted of 55 participants from each group, aged between 18 and 60 years old. Participant demographics are presented in Table 1. No differences were observed between groups in either variable.

### 2.2. Dog Breed, Characteristics, and Anthropometric Data

The weight of participating dogs was measured with a Marsden VS 250E (Marsden Equipment Co., Ltd., Rotherham, UK) digital scale. Dog neck perimeter was measured with a common anelastic tape at the base of the neck, which is the widest point of the neck. A veterinarian kindly assisted with the dog measurements.

The age of the dogs was recorded from the pets’ health booklets. Details regarding the frequency, duration, and distance of daily walks, as well as the meal volume and frequency consumed by each companion dog, were reported by the owners. Dog characteristics are presented in Table 2.

### 2.3. Anthropometry of Dog Owners and Non-Owners

Body weight and height of participants were measured in morning hours with Seca 874 portable digital scales and Seca 214 portable stadiometers, respectively (Seca GmbH & Co., Hamburg, Germany). Instruments were checked once per week for reliability and accuracy. Body mass index (BMI) was calculated as body weight (BW, kg) divided by squared height (m^2^). Weight status was defined according to the BMI cutoffs endorsed by the World Health Organization (WHO) cutoffs [29].

Body fat, as a percent of body weight, was assessed with Bioelectrical Impedance Analysis (Tanita BF-522W, Tanita UK Ltd., Middlesex, UK), and skinfold thickness was measured for all participants, at the left side of the body, with the use of a Harpenden set of calipers (Baty International, West Sussex, UK).

Waist circumference was measured with a common non-stretchable measuring tape in a horizontal plane, midway between the inferior margin of the ribs and the top of the iliac crest; hip perimeter was measured at the widest portion of the buttocks, all with the subject standing and at the end of a gentle expiration, according to the WHO [30]. Neck circumference was measured midway of the neck, between the mid-cervical spine and mid-anterior neck, at approximately 1 mm, with the subjects standing upright [31]. Among men with laryngeal prominence (Adam’s apple), the measurement was taken just below the observed prominence. The same experienced dietitians measured all anthropometric indices.

### 2.4. Physical Activity

The PAL of participants was recorded with Fitbit zip^TM^ (Fitbit Inc., San Francisco, CA, USA) wireless activity trackers for 3 consecutive days. The trackers consist of 3D-axis accelerometers that are sweat-, rain-, and splashproof and can be clipped on clothes, underwear, or pockets. Each tracker records 7 days of detailed motion data (minute by minute) and can be synchronized wirelessly to other electronic devices in order to extract data. Several studies have validated the use of Fitbit zip^TM^ [32]. The average daily steps recorded for each participant were used for the analyses.

### 2.5. Diet Quality and Knowledge

The Eating Assessment Table (EAT 2008) [33], a tabulated questionnaire, was employed to evaluate diet quality and knowledge of participants. The EAT consists of 11 domains in total, namely fruit, vegetables, legumes, meat, starchy foods, dairy and replacements, alcohol and omega-3 fatty acids, “empty” calories, cooking methods, knowledge of fats, and one miscellaneous category with information on diet diversity, portion size, meal patterns, nuts, and vitamin intake. Each of the categories can receive a maximum score of 10. The total score ranges from 0 to 100, since one of the 11 domains is subtracted. A greater EAT score is indicative of better diet quality and knowledge compared to a lower EAT score.

The Mediterranean Diet Score (MedDietScore) [34] is a widely used tool for assessing the level of adherence to the Mediterranean diet. It includes 11 core components: cereal, potatoes, fruit, vegetables, legumes, fish, red meat, poultry, dairy, olive oil, and alcohol. Each component is assigned a score from 0 to 5 based on the frequency of consumption, with higher scores reflecting greater adherence to the Mediterranean diet pattern. Higher consumption of fruit, vegetables, legumes, cereals, fish, and olive oil receives higher scores, whereas higher consumption of red meat, poultry, and dairy is scored inversely. Alcohol is scored moderately, reflecting the traditional pattern of moderate intake, particularly of wine. Greater MedDietScore values are associated with reduced cardiovascular risk [34].

### 2.6. Quality of Life

QoL was assessed using the WHOQOL-BREF questionnaire [35], an abbreviated version of the WHOQOL-100, consisting of 26 items. It produces scores in four domains: physical health, psychological, social relationships, and environment. The first two questions, which assess the overall perception of QoL and health, are not included in the domain scores. Domain scores were transformed into a linear scale from 0 to 100 according to the official scoring guidelines, with higher scores indicating better QoL. Three items were reverse-coded as per the scoring instructions.

### 2.7. Statistical Analyses

Statistical analyses were performed using SPSS version 29.0.2 (IBM Corporation, New York, NY, USA) and R studio (version 4.4.1) [36]. Normally distributed continuous variables were compared between groups using the independent samples *t*-test, while non-normally distributed variables were compared using the Mann–Whitney *U* test. To account for multiple comparisons across EAT sub-items and MedDietScore components, Bonferroni correction was applied by dividing the significance threshold (α = 0.05) by the number of tests. Medians and interquartile ranges (IQRs) were reported for non-parametric variables, while means and standard deviations (SDs) were reported for parametric variables. Bivariate correlations were performed to explore relationships between continuous variables and to assess potential multicollinearity. Correlations were computed with Spearman for non-normal data. Correlation matrices were visualized using heatmaps, highlighting the strength and direction of these relationships.

Linear regression models were constructed to examine the association of dog ownership with diet (Total EAT 2008, MedDietScore), body composition (body fat as a percentage of body weight, BF%, BMI), and QoL (WHOQOL-Bref domains), adjusted for age, sex, and total number of daily steps. Predictors were initially selected based on clinical relevance, and backward elimination was applied to remove non-significant predictors while retaining clinically important covariates. Statistical significance was set at *p* < 0.05.

## 3. Results

### 3.1. General Comparisons Between Dog Owners and Non-Owners

Dog owners had less BF (*p* = 0.009 for BF% and *p* = 0.034 for BF in kg), which persisted even after adjustment for sex, age, and BMI (*p* = 0.009), when compared to non-owners. In addition, dog owners demonstrated smaller hips circumferences (*p* = 0.001), triceps (*p* = 0.012), and subscapular (*p* = 0.003) skinfolds compared to the dog non-owners (Table 3).

No differences were observed in the number of daily steps recorded with the activity trackers between groups. Pet health condition did not appear to affect dog-walking duration, distance covered, average daily recorded steps, or the owners’ body fat (BF%). Pets living in houses with a yard were significantly heavier (*p* = 0.004), larger in size (*p* = 0.027), with greater neck circumference (*p* = 0.002), and were older (*p* < 0.001), while their daily walk duration was shorter compared to dogs without a yard (*p* = 0.004). No differences were observed for walking distance, walking frequency outside home, health condition, or number of pets cohabiting the same household.

No differences were noted between sexes among dog owners, except for pet size, which was significantly larger among men pet owners, compared to the women (*p* = 0.028).

### 3.2. Relationships Between Pet Characteristics, Owner Characteristics, and Lifestyle Measures

Dog neck circumference demonstrated a weak correlation to their owners’ biceps and triceps skinfold thickness (r = −0.327, *p* = 0.016 and r = −0.320, *p* = 0.018, respectively) and a stronger one to the distance covered during walks (r = 0.331, *p* = 0.014). What was interesting was that the dog neck circumference was not correlated to the dog’s size, but it was positively correlated with the dog’s weight (r = 0.881, *p* < 0.001). The dog’s size was negatively correlated to their owners’ triceps (r = −0.325, *p* = 0.015), sum of skinfolds (r = −0.311, *p* = 0.021), their owners’ hips (r = −0.341, *p* = 0.011), and BF and fat mass index (r = −0.357, *p* = 0.007 and r = −0.307, *p* = 0.023), respectively. The distance covered during walks was positively correlated to the psychological domain of QoL (r = 0.309, *p* = 0.022), dog weight (r = 0.346, *p* = 0.01), and size (r = 0.290, *p* = 0.032).

**Figure 1:**
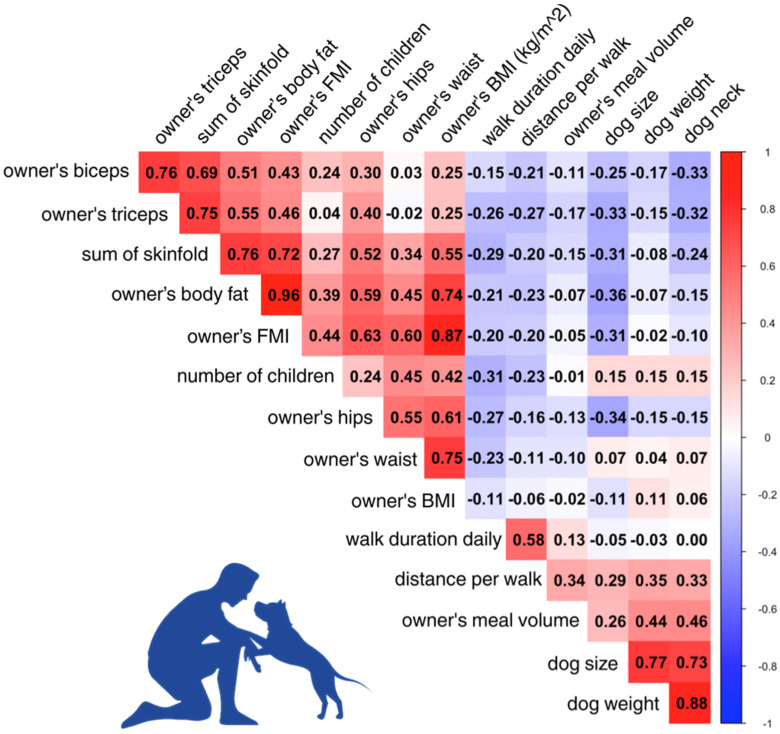
Correlation heatmap of anthropometric and health-related characteristics between dog owners and their companion dogs. BMI: body mass index; FMI: fat mass index; kg: kilogram; m: meter.

Dog weight was positively associated with the distance covered per walk (r = 0.346, *p* = 0.010) and the meal volume of the owner (r = 0.440, *p* < 0.001). Among dog owners, the psychological domain of QoL negatively correlated with the number of children the dog owners had (r = −0.346, *p* = 0.010). The daily walking duration was negatively correlated to the sum of skinfolds of the owner (r = −0.291, *p* = 0.031), hips circumference (r = −0.270, *p* = 0.031), and the number of children the dog owners had (r = −0.309, *p* = 0.022). Further details can be found in Figure 1.

When adding both groups to the correlations, the negative correlation between daily walk duration and summary of skinfolds (r = −0.291, *p* = 0.031), hips circumference (r = −0.270, *p* = 0.046), and the number of children the dog owners had (r = −0.309, *p* = 0.022) persisted. Nevertheless, among dog owners, as expected, anthropometric measures (waist, hips, skinfolds thickness) correlated strongly with BMI and BF. In addition, owner neck perimeter was negatively associated with the total EAT score (r = −0.270, *p* = 0.004) and waist circumference (r = −0.314, *p* < 0.001) and positively associated with BF (mass in kg) (r = 0.481, *p* < 0.001), waist circumference (r = 0.757, *p* < 0.001), and female sex (r = 0.279, *p* = 0.003).

The ANOVA revealed that among dog owners (*p* = 0.007), dog-walking duration was significantly different between weight status tiers, with underweight participants engaging in longer walks compared to those with obesity (*p* = 0.006) and normal body weight (*p* = 0.018).

### 3.3. Differences in Nutritional Knowledge and Diet Quality by Dog Ownership

Table 4 presents the points scored in each EAT domain between the two groups, indicating dietary practices and nutritional knowledge.

Dog owners were more familiar with the effects of trans fat consumption on the development of CVD (*p* = 0.007) but appeared less educated in recognizing starchy foods (*p* = 0.027) compared to the controls. In the two distinct categories of the EAT questionnaire, no difference was observed in the first, but dog owners scored better in the second, indicating better nutrition knowledge and dietary habits (*p* = 0.004) compared to controls. A greater total EAT score was exhibited by dog owners (*p* = 0.003), even after adjustment for the total years of attained education and BMI (*p* = 0.026). After adjusting for multiple comparisons using Bonferroni correction, differences in total EAT score, nutrition knowledge, and specific habits, and total EAT adjusted for education, remained statistically significant.

Table 5 presents the points scored in each MedDietScore category between the two groups. Non-owners of dogs showed a greater preference for potatoes (*p* < 0.001) and reported slightly higher alcohol scores (*p* = 0.009). However, after the application of Bonferroni correction for multiple comparisons, only weekly potato intake remained significant. Overall, dog ownership was not associated with meaningful differences in most Mediterranean diet components. Non-owners of dogs demonstrated a slightly greater adherence to the Mediterranean diet compared to dog owners.

### 3.4. Multivariate Regression Analyses

In the multivariate linear regression model predicting BF%, we constructed a model using dog ownership, age, sex, total daily steps, and BMI as predictors. After adjustment, dog ownership was not associated with BF (Table 6). Sex and BMI were the strongest predictors, while total daily steps showed a borderline negative association with BF%. The model explained 89.5% of the variance in BF% (Durbin–Watson = 1.882).

The first model predicting diet quality using the EAT score included the same predictors as previously mentioned. Dog ownership was independently associated with higher EAT scores. Sex was also a significant predictor, with women having better diet quality. Age, BMI, and total daily steps were not associated with total EAT score. The model explained 18.6% of the variance in diet quality (Durbin–Watson = 2.186).

Initial bivariate correlations suggested that a greater MedDietScore was associated with a higher BMI (r = 0.212, *p* = 0.03), BF (r = 0.190, *p* = 0.047), waist circumference (r = 0.224, *p* = 0.019), subscapular skinfold (r = 0.220, *p* = 0.025), and lower psychological QoL (r = −0.212, *p* = 0.026). However, when adjusting for potential confounders, including sex, BF, psychological QoL, and total steps in a multiple regression model, the MedDietScore was no longer significantly predicted by these factors (B = −1.157, *p* = 0.080), suggesting that the raw correlations were influenced by other variables. Notably, psychological QoL remained a significant independent predictor of the MedDietScore (B = −0.051, *p* = 0.048).

## 4. Discussion

The main findings of this study reveal that dog owners and controls appear to have a similar PAL and QoL, but the former exhibit improved anthropometric outcomes, diet quality, and dietary knowledge. This study associated biceps/triceps skinfolds with dog neck perimeter and dog size; the sum of skinfolds, BF, and BMI was correlated to dog size. Having a yard significantly increased pet weight, size, and neck circumference. Dog-walking duration was affected by the BMI and hips circumference of dog owners and the existence of a household yard, whereas dog-walking distance was smaller as the number of children each dog owner had increased.

Several studies have demonstrated increased PAL and a greater proportion of attainment of the recommended PAL among dog owners compared to non-dog owners [11,37]. In the present study, pedometer data indicated that although dog owners performed more steps on average than non-owners, the difference was not significant. This suggests that dog owners may be compensating for the physical activity gained from dog walking with less active behaviors during the rest of the day. These findings align with previous research showing that self-reported leisure walking was higher among dog walkers (289–383 min per week) compared to other groups (100–270 min per week), despite no differences in accelerometer-measured light or MVPA [38]. Short-term benefits, such as increased steps and sit-to-stand transitions, have been observed following dog acquisition [39], but these effects may not persist in the long term. It is possible that dog walking contributes to a perception of greater activity, or that time spent walking a dog might partially replace other forms of walking, although no differences were observed for self-reported instrumental walking or total accelerometer measures of light or MVPA [38]. Thus, while dog walking can initially motivate greater activity levels, maintaining these behavioral changes over time may require additional strategies or environmental support to ensure lasting benefit. This is especially important given that higher levels of MVPA are associated with greater objective fitness [40].

Importantly, dog ownership has also been associated with reduced sedentary behavior and increased light-intensity physical activity in adults, as measured objectively by accelerometers [41]. This is particularly relevant for older adults, as owning a dog is linked with greater daily movement, and promoting dog-friendly environments could help further increase PAL in this population [20].

The study herein demonstrated improved anthropometric outcomes among dog owners, such as a lower BF content, a smaller hips circumference, and triceps and subscapular skinfolds compared to the controls. The leaner body observed among dog owners consists of a possible epiphenomenon of dog walking, whereas the thinner skinfolds are indicative of leaner arm and subscapular muscles, which could be attributed to carrying the dog leash. Supporting this, loaner dogs have been shown to motivate walking, improve adherence to physical activity programs, and lead to health benefits such as weight loss [42]. Many dog owners cite weight loss as a motivation to walk their dog [43]; however, some studies indicate that dog ownership alone does not necessarily lead to weight loss [44], and some owners report not walking their dogs regularly [45].

The current study failed to demonstrate that dog ownership is linked to improved QoL. This lack of effect may reflect the relatively young age of the sample, which might reduce the ability to detect differences. Previous research indicates that owning a dog can reduce loneliness, enhance mental health [46], and improve multiple aspects of well-being [47]. On the other hand, negative emotions can arise when owners fail to meet their dog’s needs, encounter unwanted behaviors, or face challenges associated with caring for an aging dog, or coping with the end of a dog’s life [48].

Although the psychology and health of pet owners have been extensively researched [25], no study has examined diet adherence and knowledge. Our study showed that dog owners follow a healthier dietary regimen and attain better knowledge concerning a healthy diet compared to non-dog owners, even after the adjustment of the EAT score for years of education attained and BMI. This finding suggests that dog ownership may be associated with greater health-consciousness. However, effect sizes were generally small, limiting their clinical relevance. It remains unclear whether dog ownership directly influences eating behaviors and body composition, or whether individuals with healthier lifestyle patterns are more likely to own dogs. Further research is required to validate these associations and clarify causality. According to research, a substantial proportion of vegan/vegetarian pet owners chose a similar diet for their dogs [28], highlighting how their personal beliefs can shape the dietary choices they make for their animals [28,49]. This might explain the increased knowledge they demonstrated compared to non-owners. Nevertheless, the choice of dietary pattern may also be driven by the owners’ ethical views regarding animals [50].

Interestingly, however, non-owners exhibited greater adherence to the Mediterranean diet, as reflected by greater MedDietScore; however, this difference was primarily driven by greater potato intake. Although a previous study reported no overall differences in dietary intake between dog owners and non-owners using a semiquantitative food frequency questionnaire [51], dietary patterns among male owners appeared less favorable, with higher consumption of added fats being associated with increased BMI and a greater number of health conditions [51]. Since a lower MedDietScore has been consistently linked to a higher risk for CVD [52], the findings suggest that the lower adherence among pet owners could potentially increase CVD risk. On the other hand, aspects of pet ownership such as increased PAL and psychosocial benefits might mitigate this risk, contributing to a net protective effect on cardiovascular health [6,53]. Further research is needed to determine whether lower adherence to the Mediterranean diet might attenuate the protective effects of pet ownership on CVD outcomes.

In parallel to human obesity, canine obesity appears to grow in proportions, affecting 34–59% of dogs today [54,55,56]. Dogs and their owners share the same ‘family food environment’ [57,58], and this is why several studies have associated pet anthropometric characteristics with their owners [24]. Additionally, evidence suggests that owners’ personal dietary habits may influence not only their own health behaviors but also the feeding choices they make for their dogs, reflecting a broader pattern of health-conscious decision-making [49]. The present study revealed a connection between pet weight and the owners’ meal volume. In addition, dog neck circumference was negatively associated with their owners’ biceps and triceps skinfolds, and dogs’ size was negatively correlated to the sum of skinfolds, as well as the biceps/triceps of the owner, BF, and fat mass index. With neck perimeter being associated with dog size, it is possible that a greater dog neck perimeter requires increased arm strength during dog walking, resulting in leaner upper arms for the dog owners. The observed associations may reflect lifestyle patterns, such as shared physical activity or feeding habits, but further research is required to confirm these preliminary findings.

Several factors affecting dog walking have been proposed in the literature, including pet health, the existence of a yard, elevated owner BMI, or even the number of children inhabiting the household. Cutt and associates [9] suggested that a dog’s health positively affects dog walking, while others reported a lack of associations [59,60,61] in accordance with the present findings. According to Robertson [62], dog walking is directly associated with the pet’s weight, as seen in the results herein, with larger dogs requiring considerably more exercise. Studies have shown that overweight dogs are exercised less frequently [62,63], and this has been verified even with the use of pedometers on the dogs [64]; however, canine weight status was not assessed in the present study. In addition, owners often fail to correctly identify the body condition of their dogs, and despite daily walks, many dogs still have excessive BF [65]. Evidence suggests that increasing the number of walks per week is more effective for weight loss than extending the duration of individual walks, and that moderate, frequent walks are preferable for weight management and stress reduction in overweight dogs compared to fewer long walks [66]. Conversely, current results indicate that the existence of a yard limits dog-walking duration, whereas other researchers have proposed that the actual size of the yard might be the factor reducing dog walking [9]. Additionally, yards appear to affect pet weight status, as the findings herein showed increased pet size, weight, and neck perimeter among dogs living in houses with backyards. Among the identified factors limiting dog walking in our study was the increased number of children each dog owner had. In contrast to the present results, Japanese dog owners [67] with children exhibited increased dog walking compared to those living alone; however, that study included dog walking from all family members and not only from one caregiver. Finally, the present results showed that dog-walking duration is reduced as the owner’s weight status tier is increased. According to some researchers, it is actually dog walking, as opposed to dog ownership, that appears to be associated with a lower incidence of obesity [12,68].

The present study is biased by the small sample of participants, the local representativeness, and the lack of age- and sex-matched controls. In addition, the relatively young age of the sample and the recruitment from only one city may introduce selection bias and limit the generalizability of the findings to broader populations. The use of self-reported questionnaires to assess food intake may be subject to recall bias. Furthermore, the cross-sectional design of the study does not allow for causal inference. However, the use of accelerometers was limiting recruitment, as many possible participants refused/failed to use the devices for three consecutive days. Nevertheless, we acknowledge that the relatively short monitoring period may not fully reflect habitual PAL. Additionally, the recruitment of a greater sample would introduce further bias in the results by increasing data collection duration, which would, in fact, be affected by the seasonal weather change known to alter PAL. However, the present study is unique in both the use of accelerometers as well as the evaluation of nutritional knowledge and dietary behavior.

## 5. Conclusions

The present study showed that, although PAL did not differ markedly between dog owners and non-owners, dog ownership was associated with a leaner body composition. Dog owners also appeared to possess greater nutritional knowledge and adhered to healthier dietary patterns, as shown by the EAT 2008, reflecting a more health-conscious lifestyle. Given the relatively young age of the sample and the local recruitment, further research should include a more diverse population, employ longer follow-up periods, and consider evaluating QoL and adherence to the Mediterranean diet.

## Figures and Tables

**Table 1:** Sample demographics.

	Dog Owners (*n* = 55)	Non-Owners (*n* = 55)
Sex (men/women) (*n*)	22/33	19/36
Age (years)	31.6 ± 11.7	32.8 ± 11.9
BMI (kg/m^2^)	23.3 ± 3.3	24.7 ± 3.9
Weight Status (underweight/normoweight/overweight/obese) (*n*)	3/41/8/3	2/23/14/7
Education (primary/secondary/tertiary/postgraduate) (*n*)	2/10/37/6	1/14/40/0
Marital status (single/married/divorced/widow)	40/11/2/2	32/22/1/0
Number of children (0/1/2/3) (*n*)	38/3/11/3/0	32/2/12/9
Occupation (student/employee/unemployed/household/retired/entrepreneur) (*n*)	16/19/5/1/1/13	15/26/4/1/3/6

BMI: body mass index; kg: kilogram; m: meter; *n*: number of patients in each category.

**Table 2:** Breeds and characteristics of participating dogs.

**Dog breed** (*n*)	Akita/Boxer/Brack/Canis/Cocker/German Sheppard/Golden Retriever	1/3/3/1/3/4/2
	Greek Kokoni/Greyhound/Husky/Jack Russell/Labrador/Maltese	1/1/1/2/1/3
	Pincher/Pointer/Puggle/Tsow tsow/Yorkshire/Mixed breeds	1/1/1/1/3/1/21
**Number of dogs** in household (1/2/3) (*n*)		50/3/2
**Dog breed size** (large/medium/small) (*n*)		13/24/18
**Dog weight** (kg)		15.9 ± 9.1
**Dog age** (years)		4.7 ± 2.9
**Dog neck perimeter** (cm)		31.5 ± 10.0
**Able-bodied dogs** (*n*)		55
**Apparently healthy dogs** (*n*)		53

cm: centimeter; kg: kilogram; *n*: number of patients in each category.

**Table 3:** General comparisons between dog owners and non-owners.

	Variables	Dog Owners (*n* = 55)	Non-Owners (*n* = 55)	Effect Size	*p*-Value
Body fat	Body fat (% body weight) *	24.0 ± 6.0	27.1 ± 6.7	−0.46	0.009
	Body fat (kg) adjusted for sex, age, and BMI *	24.1 ± 5.9	27.0 ± 6.7	−0.46	0.009
	Body fat (kg) ^♮^	15.8 (7.0)	17.66 (7.18)	−0.34	0.034
Perimeters	Waist circumference (cm) ^♮^	76.0 (13.0)	79.0 (11.3)	−0.05	NS
	Neck circumference (cm) ^♮^	34.0 (5.0)	33.40 (5.5)	−0.22	NS
	Hips circumference (cm) ^♮^	95.5 (11.5)	103.00 (9.8)	−0.04	<0.001
Skinfolds	Biceps skinfold (mm) ^♮^	10.0 (6.0)	9.40 (5.3)	−0.28	NS
	Triceps skinfold (mm) *	16.3 ± 6.0	18.9 ± 5.8	−0.01	0.012
	Subscapular skinfold (mm) ^♮^	12.4 (5.3)	15.6 (5.5)	−0.001	0.003
	Suprailiac skinfold (mm) ^♮^	18.1 (11.8)	20.5 (10.2)	−0.15	NS
Steps	Average steps/day ^♮^	8352 (5301)	7122 (4116)	−0.02	NS
	Average steps/day adjusted for age ^♮^	8541 (795)	8335 (1015)	−0.06	NS
	Average steps/day adjusted for age and BMI ^♮^	8467 (812)	8494 (1072)	−0.01	NS
	Average dog-walk duration/day (min)	50.7 ± 32.1	-		
	Average dog-walk distance/day (m)	1091.8 ± 647.5	-		
QoL	Physical domain of QoL ^♮^	57.1 (14.3)	53.6 (14.3)	−0.07	NS
	Psychological domain of QoL ^♮^	62.5 (12.5)	62.5 (16.6)	0.00	NS
	Social relations domain of QoL ^♮^	66.7 (25.0)	75.0 (25.0)	−0.07	NS
	Environmental domain of QoL ^♮^	59.4 (15.7)	68.8 (18.8)	−0.07	NS

* tested with the independent samples *t*-test (mean ± standard deviation); ♮ tested with the Mann–Whitney U test (median (interquartile ranges). Effect sizes for *t*-tests are reported as Cohen’s d (negative values indicate lower values in dog owners). Effect sizes for Mann–Whitney U tests are calculated as r = Z/√N. NS: Not significant; BMI: body mass index.

**Table 4:** Scoring of each Eating Assessment Table (EAT 2008) question in each group.

Q	Category	Dog Owners (*n* = 55)	Non-Owners (*n* = 55)	Effect Size	*p*-Value *
1	Fruit ^♮^	6.0 (4.0, 6.0)	4.0 (2.0, 6.0)	−0.10	NS
2	Vegetables ^♮^	4.0 (4.0, 6.0)	4.0 (4.0, 4.0)	−0.14	NS
3	Legumes ^♮^	4.0 (4.0, 4.0)	4.0 (4.0, 4.0)	−0.02	NS
4	Cooking methods ^♮^	10.0 (8.0, 10.0)	8.0 (8.0, 10.0)	−0.16	NS
5a	Fat content of meat intake ^♮^	4.0 (2.0, 4.0)	2.0 (2.0, 4.0)	−0.23	0.017
5b	Weekly meat intake ^♮^	3.0 (3.0, 3.0)	3.0 (2.0, 3.0)	−0.07	NS
5c	Average meat portion ^♮^	2.0 (1.0, 2.0)	2.0 (1.0, 2.0)	−0.18	NS
6a	Whole grain vs. processed ^♮^	2.0 (2.0, 4.0)	2.0 (0.0, 2.0)	−0.15	NS
6b	Recognition of starchy foods ^♮^	2.0 (1.0, 3.0)	3.0 (2.0, 3.0)	−0.21	0.027
6c	Portion control ^♮^	2.0 (2.0, 3.0)	2.0 (2.0, 3.0)	−0.13	NS
7a	Total dairy ^♮^	2.0 (0.0, 2.0)	2.0 (0.0, 2.0)	−0.11	NS
7b	High-fat dairy ^♮^	0.0 (−1.0, 0.0)	0.0 (0.0, 0.0)	−0.01	NS
7c	Low-fat dairy ^♮^	0.0 (0.0, 2.0)	0.0 (0.0, 0.0)	−0.21	0.027
8a	Knowledge of nutritional labels concerning fat ^♮^	2.0 (0.0, 2.0)	2.0 (0.0, 2.0)	−0.14	NS
8b	Knowledge of trans fats in increasing CVD risk ^♮^	2.0 (0.0, 2.0)	0.0 (0.0, 2.0)	−0.26	0.007
8c	Distinction between MUFA, PUFA, and SFA ^♮^	2.0 (0.0, 2.0)	2.0 (0.0, 2.0)	−0.4	NS
8d	Ability to calculate % energy from fat ^♮^	0.0 (0.0, 2.0)	0.0 (0.0, 0.0)	−0.16	NS
8e	Regularly read nutrition labels to estimate fat intake ^♮^	0.0 (0.0, 2.0)	0.0 (0.0, 2.0)	−0.04	NS
9a	Weekly alcohol intake ^♮^	0.0 (0.0, 1.5)	0.0 (0.0, 0.0)	−0.34	0.001
9b	Fish intake ^♮^	1.0 (1.0, 2.0)	1.0 (1.0, 2.0)	−0.02	NS
9c	Omega-3 fatty acid intake ^♮^	1.0 (0.0, 2.0)	1.0 (0.0, 3.0)	−0.05	NS
10a	Diversity ^♮^	2.0 (2.0, 2.0)	2.0 (2.0, 2.0)	−0.03	NS
10b	Portion control ^♮^	2.0 (0.0, 2.0)	2.0 (0.0, 2.0)	−0.15	NS
10c	Nuts intake ^♮^	2.0 (0.0, 2.0)	0.0 (0.0, 2.0)	−0.15	NS
10d	Vitamin supplements intake ^♮^	0.0 (0.0, 0.0)	0.0 (0.0, 0.0)	−0.07	NS
10e	Grazing ^♮^	2.0 (0.0, 2.0)	0.0 (0.0, 2.0)	−0.02	0.008
11a	Empty calories from drinks ^♮^	0.0 (−2.0, 0.0)	0.0 (−2.0, 0.0)	−0.25	NS
11b	Empty calories from salty snacks ^♮^	0.0 (0.0, 0.0)	0.0 (−2.0, 0.0)	−0.14	NS
11c	Empty calories from 1 dessert/day ^♮^	0.0 (−2.0, 0.0)	0.0 (−2.0, 0.0)	−0.03	NS
11d	Empty calories from 2 desserts/day ^♮^	0.0 (0.0, 0.0)	0.0 (0.0, 0.0)	−0.12	NS
11e	Empty calories from sweet snacks ^♮^	0.0 (0.0, 0.0)	0.0 (−2.0, 0.0)	−0.04	NS
1–4	Food groups intake and cooking methods *	23.1 ± 5.2	21.5 ± 4.3	0.34	0.004
5–11	Nutrition knowledge and specific habits *	29.3 ± 9.2	24.2 ± 9.1	0.55	0.002
Total EAT score (raw) *		52.2 ± 12.3	45.7 ± 12.0	0.53	0.003
Total EAT score (adjusted for education and BMI) *		49.6 ± 2.6	48.3 ± 3.3	−0.43	0.026

* tested with the independent samples *t*-test (mean ± standard deviation); ♮ tested with the Mann–Whitney U test (median (interquartile ranges). Effect sizes for *t*-tests are reported as Cohen’s d (negative values indicate lower values in dog owners). Effect sizes for Mann–Whitney U tests are calculated as r = Z/√N. BMI: body mass index; Q: question; EAT: Eating Assessment Table; NS: not significant; CVD: cardiovascular disease; MUFA: mono-unsaturated fatty acids; PUFA: poly-unsaturated fatty acids; SFA: saturated fatty acids.

**Table 6:** Multivariate analyses of body fat (% of body weight), EAT score, and MedDietScore.

BF (% of Body Weight)					EAT Score					MedDietScore				
Variable	B	β	95%Cl	*p*-Value	Variable	B	β	95%CI	*p*-Value	Variable	B	β	95%CI	*p*-Value
Dog owner	−0.08	−0.01	−0.97, 0.81	0.862	Dog owner	6.29	0.25	1.8, 10.8	0.007	Dog owner	−1.78	−0.17	−2.5, 0.1	0.08
Age	0.03	0.05	−0.02, 0.07	0.21	Age	0.02	0.02	−0.2, 0.2	0.87	Sex (w)	1.01	0.17	−0.2, 2.5	0.09
Sex (w)	7.01	0.5	6.0, 8.0	<0.001	Sex (w)	8.01	0.31	3.0, 13.0	0.002	BF (kg)	0.04	0.08	−0.05, 0.12	0.40
Total steps	−0.000035	−0.061	−0.00007, 0.000001	0.065	Total steps	0.00006	0.06	−0.0001, 0.0002	0.51	Psychological QoL	−0.051	−0.19	−0.1, 0.0	0.048
BMI	1.68	0.91	1.5, 1.9	<0.001	BMI	−0.34	−0.10	−1.1, 0.4	0.36	Total steps	−0.000002	−0.01	0.0, 0.0	0.926

B = unstandardized regression coefficient; BF: body fat; BMI: body mass index; CIs: confidence intervals; EAT: Eating Assessment Table; kg: kilogram; MedDietScore: Mediterranean Diet Score; w: women.

**Table 5:** Scoring of each MedDietScore question in each group.

Category	Dog Owners (*n* = 55)	Non-Owners (*n* = 55)	Effect Size	*p*-Value *
Cereal ^♮^	1.0 (1.0–2.0)	1.0 (0.0–2.0)	−0.15	NS
Potatoes ^♮^	1.0 (1.0–2.0)	3.0 (2.0–4.0)	−0.62	<0.001
Fruit ^♮^	2.0 (1.0–3.0)	2.0 (1.0–3.0)	−0.12	NS
Vegetables ^♮^	2.0 (2.0–3.0)	2.0 (2.0–2.0)	−0.16	NS
Legumes ^♮^	2.0 (2.0–2.0)	2.0 (2.0–2.0)	0.00	NS
Fish ^♮^	1.0 (1.0–2.0)	2.0 (1.0–2.0)	−0.01	NS
Red meat ^♮^	4.0 (4.0–5.0)	4.0 (4.0–5.0)	−0.13	NS
Poultry ^♮^	5.0 (4.0–5.0)	5.0 (4.0–5.0)	−0.04	NS
Dairy ^♮^	4.0 (4.0–5.0)	5.0 (4.0–5.0)	−0.13	NS
Olive oil ^♮^	5.0 (5.0–5.0)	5.0 (4.0–5.0)	−0.18	NS
Alcohol ^♮^	5.0 (4.0–5.0)	5.0 (5.0–5.0)	−0.25	0.009
Total MedDietScore *	33.8 ± 4.0	35.1 ± 2.7	−0.40	0.018

MedDietScore: Mediterranean Diet Score. * tested with the independent samples *t*-test (mean ± standard deviation); ♮ tested with the Mann–Whitney U test (median (interquartile ranges). Effect sizes for *t*-tests are reported as Cohen’s d (negative values indicate lower values in dog owners). Effect sizes for Mann–Whitney U tests are calculated as r = Z/√N.

## Data Availability

The original contributions presented in this study are included in the article. Further inquiries can be directed to the corresponding author.

## Abbreviations

The following abbreviations are used in this manuscript:

BF%	Body fat percentage
BMI	Body mass index
CVD	Cardiovascular disease
EAT	Eating Assessment Table
IQR	Interquartile ranges
MedDietScore	Mediterranean Diet Score
MVPA	Moderate-to-vigorous physical activity
PAL	Physical activity level
QoL	Quality of life
SD	Standard deviation
WHO	World Health Organization
WHOQOL-BREF	World Health Organization Quality of Life-brief
